# The unique contribution of Schizotypal personality subscales to psychotic-like experiences and social-personality factors in Hong Kong community youths

**DOI:** 10.3389/fpsyt.2026.1590707

**Published:** 2026-03-03

**Authors:** Melody Miriam So, Stephanie Ming Yin Wong, Yi-nam Suen, Sherry Kit Wa Chan, Edwin Ho Ming Lee, Eric Yu Hai Chen, Christy Lai Ming Hui

**Affiliations:** 1 Department of Psychiatry, School of Clinical Medicine, Lee Ka Shing Faculty of Medicine, The University of Hong Kong, Hong Kong, Hong Kong SAR, China; 2 Department of Social Work and Social Administration, The University of Hong Kong, Hong Kong, Hong Kong SAR, China; 3 School of Nursing, LKS Faculty of Medicine, The University of Hong Kong, Hong Kong, Hong Kong SAR, China; 4 The State Key Laboratory of Brain and Cognitive Sciences, The University of Hong Kong, Hong Kong, Hong Kong SAR, China; 5 Orygen, The National Centre of Excellence in Youth Mental Health, University of Melbourne, Melbourne, VIC, Australia; 6 Centre for Youth Mental Health, The University of Melbourne, Melbourne, VIC, Australia

**Keywords:** personality, psychotic-like experience (PLE), schizotypy, social anxiety, youth and adolescence

## Abstract

**Introduction:**

Schizotypal personality is a multifaceted construct that can be presented as the subscription to unusual thinking and behavior, few close relationships, and paranoia. The brief form of the Schizotypal Personality Questionnaire indexed these tendencies into Interpersonal, Cognitive-Perceptual, and Disorganization subscales. The current study aims to validate the three-factor structure of self-report subclinical schizotypy traits among an epidemiological youth sample in Hong Kong and their relationship with psychotic-like experiences, personality traits, and social factors.

**Methods:**

3186 participants (58.3% female) between the ages of 15 and 25 (mean=19.8, SD = 2.8) completed a self-administered questionnaire package comprised of the Schizotypal Personality Questionnaire-Brief form, the Prodromal Questionnaire-Brief, the Liebowitz Social Anxiety Scale, and the Big Five Inventory. Factor analysis was conducted on schizotypal items, and the association between outcomes was tested using hierarchical linear regression.

**Results:**

Confirmatory Factor Analysis suggested that the three-factor model was a moderate-to-good fit. The Interpersonal subscale was associated with all outcomes and had higher explanatory power on social outcomes. Cognitive-Perceptual and Disorganization subscales had higher explanatory power on psychotic-like experiences.

**Discussion:**

The study highlights the replicability of subclinical schizotypy traits in Hong Kong youths. Each of the three factors had overlapping and unique associations with psychopathological and social-personality outcomes that can predispose individuals to mental ill health.

## 1 Introduction

Schizotypy is characterized by odd or eccentric beliefs, speech, and behaviors that are temporally stable (1). Subclinical schizotypy traits can generally be categorized into three domains: Interpersonal deficits, Cognitive-Perceptual deficits, and Disorganization (2). These domains correspond to negative, positive, and disorganized schizotypy (3). Some researchers recognized schizotypal features as subclinical psychotic experiences on the spectrum of schizophrenia, while others separated the condition as latent traits related to the genetic vulnerability of schizophrenia (4, 5). Screening of schizotypal features could inform current functioning, as well as familial-genetic and environmental risk factors (6, 7).

A huge body of research has established an association between schizotypy and psychotic-like experiences (PLEs), such that individuals with higher schizotypal traits reported more PLEs (8, 9). In particular, experience sampling methodological studies found that positive schizotypy specifically predicted PLEs, whereas negative schizotypy was associated with diminished positive affect and social disinterest (10, 11). A review of the dimensionality of schizotypy supports the view that positive schizotypy in healthy individuals was more relevant to PLEs, independently of cognitive disorganization; whereas negative schizotypy was more informative of life quality (12). Separately, disorganization was more closely linked to cognitive deterioration and associative looseness than the other factors (13, 14). Given that the relevance of schizotypy subtypes to social and cognitive domains differed, the subtypes may uniquely contribute to different experiences on the schizophrenic spectrum. However, limited studies have done a direct comparison between the predictive power of schizotypy traits on psychopathological outcomes.

Subclinical schizotypy traits also feature a persistent deficit in social functioning, reflected by difficulties in forming and maintaining relationships (15, 16). It is not uncommon for schizotypal patients to be diagnosed with social anxiety disorder, autistic spectrum disorder, and antisocial personality disorder (1). Individuals with high levels of schizotypy were found to exhibit impaired facial emotion recognition (17) and higher referential thinking than social anxiety patients (18). Social anxiety has been consistently identified in the structure of the Schizotypal Personality Questionnaire (SPQ) and its brief form (SPQ-B) (19–21). Notably, most studies have used a college sample to investigate social anxiety in schizotypy (19, 22, 23), with few done in a representative sample (21) or an Asian sample (24). Validation of a social anxiety component in schizotypy among Chinese youths would confirm the generalizability of the three-factor structure in predicting mental well-being.

The suboptimal functioning that is associated with subclinical schizotypy traits may reflect on the measure of normal personality traits. One common taxonomy of personality that was compared to schizotypy is the Big Five Personality, comprised of agreeableness, conscientiousness, extraversion, neuroticism, and openness to experience (25). While both represent personality constructs, schizotypy specializes in psychosis-related tendencies, whereas the Big Five covers general characteristic patterns. Out of the five traits, openness has garnered attention for its potential to account for the creativity and acceptance of new experiences in positive schizotypy that is characterized by unusual beliefs (26–29). Some researchers also suggested that low levels of trust, warmth, and gregariousness in negative schizotypy might reflect reduced agreeableness, reduced extraversion, and increased neuroticism (30). It is less clear whether other personality facets, such as conscientiousness and disorganized schizotypy, would interact similarly.

Based on previous research, the multifaceted nature of schizotypy suggests each dimension (Interpersonal, Cognitive-Perceptual, and Disorganization) can have overlapping as well as distinct influences on mental health. Understanding each schizotypy dimension relates to psychopathological and social expression may provide insight into the heterogeneity of impairments experienced by the individual (31). While the relationship between schizotypy and PLEs has been established (9), the current study further compared subtypes in the prediction of PLEs. This may identify the most relevant trait to specifically improve the sensitivity of screening for psychosis transition in youths. Similarly, the unique predictive power of subtypes on social and personality outcomes may highlight the individual differences in prognosis. A brief measurement of schizotypy, such as the SPQ-B, offers a low-demand and fast screening of indicative risk markers, especially in a quick-paced city like Hong Kong. Together, this warrants the investigation of the three-factor SPQ-B model and its association with psychotic-like experiences, social anxiety, and personality factors in a representative Hong Kong youth sample.

The current study aimed to 1) validate the three-factor SPQ-B model, 2) confirm components of PLEs and social anxiety in schizotypy, and 3) explore the relationship with normal personality traits. We hypothesized that a three-factor schizotypal construct that corresponds to the Cognitive-Perceptual, Interpersonal, and Disorganization items would be negatively associated with agreeableness, conscientiousness, openness, and extraversion, but positively associated with PLEs, PLE distress, trait neuroticism, and social anxiety. We expected the Interpersonal subscale to have higher explanatory power on social-personality outcomes, whereas Cognitive-Perceptual deficit and Disorganization would contribute more to the explanation of PLE outcomes.

## 2 Methods

### 2.1 Participants

The present study was conducted as part of the Hong Kong Youth Epidemiological Study (HK-YES), which was a territory-wide household-based epidemiological study aimed at evaluating the mental health conditions of the local youth population aged 15–24 years. Young people within this age range were recruited by mailing invitation letters to random residential addresses with stratification by geographic district and housing type (C. S.-M. 32; S. M. Y. 33). Participants who do not reside in Hong Kong are excluded. The excluded group had higher percentages of male and non-Chinese ethnicity, but there were no significant differences in age and education level (**Supplementary Material I**). Recruitment was conducted from May 2019 to July 2022.

The response rate was 66.5% with a total of 3460 participants who provided consent to the study, and 3186 (92.1%) completed data on the following variables of interest were included in the present analysis: age, sex, educational level, Schizotypal Personality Questionnaire-Brief form (SPQ-B), Prodromal Questionnaire-Brief (PQB-21), Big Five Inventory (BFI), Liebowitz Social Anxiety Scale (LSAS). All participants provided written informed consent or parental consent for those under 18 years of age.

The current sample had a mean age of 19.84 years (SD = 2.79), and 58.3% were female (**Supplementary Material I**). Three participants did not provide information on their first language. Among the 3029 participants, 97.6% were Chinese, and 93.1% reported Cantonese as their first language. There were no differences between those who completed pre-tertiary (i.e., before diploma) and tertiary education in Cognition-Perceptual (M = 2.22, SD = 1.80; M = 2.13, SD = 1.87; *p* = .176), Interpersonal (M = 2.84, SD = 2.25; M = 2.91, SD = 2.37; *p* = .383), and Disorganization (M = 1.24, SD = 1.53; M = 1.25, SD = 1.53; *p* = .928) subscales. The study was approved by the Institutional Review Board of the University of Hong Kong/Hospital Authority Hong Kong West Cluster (Reference number: UW19-017) and carried out in accordance with Good Clinical Practice and the Declaration of Helsinki.

### 2.2 Measures

#### 2.2.1 Schizotypal personality traits

SPQ-B is a 22-item dichotomous (yes=1, no=0) scale designed to measure a three-factor structure of schizotypal personality: Cognitive-Perceptual deficit, Interpersonal deficit, and Disorganization (34). The 8-item Interpersonal subscale (items 1, 7, 11, 14, 15, 18, 21, 22) measures social anxiety, lack of friends, blunted affect, and paranoid ideation and showed good internal consistency in this sample (*ω* = .780). The Cognitive-Perceptual subscale contains 8 items (items 2, 4, 5, 9, 10, 12,16, 17) that measure content reflecting ideas of reference, magical thinking, unusual perceptual experiences, and paranoid ideation (*ω* = .655). A fourth factor, Paranoid Ideation, can be deduced from the items of these two subscales (items 7, 9, 14, 15, 17). This factor was found in a Chinese non-clinical sample (35) and is expected to be related to both constructs (36). There are 6 items to the Disorganization subscale (items 3, 6, 8, 13, 19, 20), which measures odd behavior and odd speech (*ω* = .730). A higher score indicates a stronger schizotypal personality trait. SPQ-B has been validated and used in Chinese samples (37, 38).

#### 2.2.2 Psychotic-like experience symptoms and distress

PQB-21 contains 21 items that measure the presence of PLE symptoms on a dichotomous yes-no scale (*ω* = .830) and the level of distress associated with the symptoms (*ω* = .851) on a scale of 1 (strongly disagree) to 5 (strongly agree). The scale showed acceptable item-total correlation of the symptom (*rs =* 0.32 – 0.60, *ps* <.001) and distress subscales (*rs =* 0.30 – 0.62, *ps* <.001). A higher score suggests a higher level of PLE symptoms and distress, respectively. The cut-off scores are 7 for the PLE subscale and 24 for the distress subscale (39).

#### 2.2.3 Big Five personality traits

BFI (40) is a 44-item scale used to evaluate agreeableness (9 items; *ω* = .677), conscientiousness (9 items; *ω* = .784), extraversion (8 items; *ω* = .813), neuroticism (8 items; *ω* = .812), and openness (*ω* = .802). Participants answer on a Likert scale ranging from 1 (strongly disagree) to 5 (strongly agree). 16 items are reverse-scored and summed to calculate five subscale scores. Higher scores indicate greater expression of the respective personality trait, as previous studies in Chinese samples showed (25, 41).

#### 2.2.4 Social anxiety

LSAS (42) consists of 24 items that assess social anxiety symptoms on two subscales: fear (*ω* = .936) and avoidance (*ω* = .929) of social situations. Each item is rated on a scale of 0 (never) to 4 (usually) and summed. A higher score on each subscale represents a higher degree of fear and a higher likelihood of avoidance, respectively.

### 2.3 Statistical analysis

Analyses were conducted with R (packages *lavaan*) and SPSS (version 27). The factor structure was evaluated with confirmatory factor analysis (CFA). As the SPQB items are ordinal (yes-no), we employed categorical CFA with the Weighted Least Squares Mean and Variance adjusted (WLSMV) estimator. All 22 items were treated as ordered categorical variables, with freely estimated thresholds for each response category. Latent factor variances were fixed to 1.0 to produce standardized solutions. The model was specified as a three-factor model corresponding to the Cognitive-Perceptual, Interpersonal, and Disorganized dimensions.

A significant *χ²* in CFA usually suggests poor model fit, however, a large sample size in this study can increase the sensitivity of *X^2^* to detect smaller effects. A model with a Comparative Fit Index (CFI) above 0.95, a Root Mean Square Error of Approximation (RMSEA) below 0.06, Standardized Root Mean Square Residual (SRMR) below 0.08, and Tucker-Lewis Index (TLI) above 0.90 is considered a good fit (43). Scaled values are reported for CFA model fit indices. A factor loading above 0.5 suggests a strong relationship between the item and underlying factor.

A partial correlation with age, sex (female as 1, male as 2), and education level as control variables was performed. To evaluate and compare the unique contributions of each schizotypy subscale, we performed a sequential dominance analysis (a form of hierarchical partitioning). After controlling for demographic covariates (sex and age) in the first block, the three subscales were systematically permuted and entered in the final block across all possible sequences. For each predictor, we calculated its average incremental increase in R² (ΔR²) across these permutations. The ΔR² associated with each subscale when entered last represents its unique explanatory power for the outcome, controlling for all other predictors. It should be noted that, like all dominance-based methods, these estimates are conditional on the specific predictor set and do not fully orthogonalize shared variance among correlated predictors. Consequently, findings are reflective of conditional contributions and should not be interpreted as independent effect sizes.

To account for multiple comparisons across all statistical tests, we applied False Discovery Rate (FDR) correction using the Benjamini-Hochberg procedure with *α* = .05 to the linear regression models. This controls the expected proportion of false discoveries rather than the familywise error rate.

## 3 Results

### 3.1 Evaluation of the three-factor structure

CFA of the three-factor model showed a moderate-to-good fit by conventional criteria: (*χ²*(206, 3186) = 2039.51, *p* < 0.001, CFI = 0.933, RMSEA = 0.053, SRMR = 0.071, TLI = 0.925). Factor correlation revealed significant positive relationship between the three factors (see **Table 1**). A four-factor model was also tested in reference to the Paranoid Ideation factor (items 7, 9, 14, 15, 17). Model comparison revealed no significant improvement for the four-factor model (*χ*²(203, 3186) = 2045.56, *p* < 0.001, CFI = 0.933, TLI = 0.923, RMSEA = 0.053, SRMR = 0.071) over the three-factor model, Δ*χ*²(3) = 6.789, p = 0.079 (see **Supplementary Material II**). Given the non-significant improvement and the principle of model simplicity, the three-factor model was retained.

**Table 1:** Standardized loading and factor correlation of the three-factor SPQ-B model using CFA.

		Factor loading		*h^2^*
Items		*Λ*	95% CI	
Interpersonal				
1.	Sometimes other people think that I am detached or distant	0.707	0.671, 0.742	0.500
7.	I feel that I must be vigilant even with my friends #	0.633	0.595. 0.672	0.401
11.	I don’t feel at all at ease when I am around people I don’t know	0.594	0.556, 0.633	0.353
14.	I think it’s better that people don’t know too much about me #	0.726	0.694, 0.758	0.527
15.	I tend to stay withdrawn when I am in social situations #	0.810	0.783, 0.836	0.656
18.	I have the feeling that I can’t be close to people	0.822	0.796, 0.847	0.675
21.	I don’t feel comfortable when I talk to people I don’t know well	0.783	0.755, 0.812	0.614
22.	I tend to keep my feelings to myself	0.644	0.605, 0.684	0.415
Cognitive-Perceptual				
2.	I happen to feel an unseen force or presence around me	0.612	0.559, 0.665	0.375
4.	I am sometimes convinced that other people are able to guess what I think	0.360	0.308, 0.413	0.130
5.	It happens to me that certain objects or ordinary situations have a special significance for me	0.620	0.579, 0.661	0.384
9.	It often happens to me to see hidden threats or derogatory remarks in what other people say or do #	0.745	0.707, 0.782	0.554
10.	When I go shopping I have the feeling that people notice me	0.639	0.596, 0.682	0.408
12.	It happened to me to have special experiences about astrology, premonition, unidentified flying objects, extrasensory perception, or the sixth sense	0.378	0.326, 0.429	0.143
16.	Sometimes, I'm suddenly distracted by distant sounds to which usually I don't pay much attention.	0.685	0.644, 0.725	0.469
17.	I often must be vigilant for other people not to take advantage of me #	0.529	0.483, 0.576	0.280
Disorganization				
3.	Sometimes other people comment on my behavior particularities or my unusual habits	0.669	0.628, 0.710	0.448
6.	Some people believe I am very odd/bizarre	0.830	0.803, 0.857	0.689
8.	Some people consider my way of speaking vague or not too clear	0.774	0.743, 0.805	0.599
13.	I sometimes use words in an unusual way	0.593	0.593, 0.647	0.351
19.	I am a strange or unusual person	0.799	0.772, 0.826	0.639
20.	It’s difficult for me to make other people understand what I want to say	0.741	0.705, 0.776	0.549
Factor Correlations		*R*	*95% CI*	
	Cognitive-Perceptual x Interpersonal	0.667	0.631, 0.703***	
	Cognitive-Perceptual x Disorganization	0.748	0.713, 0.783***	
	Interpersonal x Disorganization	0.830	0.805, 0.855***	

SPQ-B, Schizotypal Personality Questionnaire-Brief; N, 3186; ***p < .001.

#Paranoid Ideation items are expected to load on both Cognitive-Perceptual and Interpersonal factors.

Given suboptimal loadings for items 4 (*λ* = 0.360) and 12 (*λ* = 0.378) on the Cognitive-Perceptual factor, we conducted a sensitivity analysis excluding these items. The reduced 20-item model showed comparable fit (*χ²*(167, 3186) = 1790,58, *p* < 0.001, CFI = 0.938, RMSEA = 0.055, SRMR = 0.071, TLI = 0.930) to the full model with improved Cognitive-Perceptual psychometrics (average *λ* = 0.632 vs. 0.571; minimum *λ* = 0.525 vs. 0.360). We retain the full scale to preserve content validity while noting that Cognitive-Perceptual findings represent conservative estimates due to measurement attenuation.

### 3.2 Relationship with clinical variables and normal personality traits

When controlling for age, sex, and education level, the three factors were positively correlated with PLEs, social anxiety, and neuroticism, but negatively correlated with agreeableness, conscientiousness, extraversion, and openness. A very weak but significant negative correlation was found between Interpersonal deficits and openness (**Table 2**).

**Table 2:** Correlations between the three SPQ-B subscales and PLEs (PQB-21), social anxiety (LSAS), and Big Five personality traits (BFI), controlling for age, sex, and education level.

			SPQ-B subscales					
Variables	Mean	SD	Interpersonal		Cognitive- Perceptual		Disorganization	
			*r*	*p*	*r*	*p*	*r*	*p*
Schizotypal personality (SPQ-B)								
Interpersonal	2.87	2.29	/	/	/	/	/	/
Cognitive-Perceptual	2.19	1.89	0.458	<0.001	/	/	/	/
Disorganization	1.25	1.53	0.608	<0.001	0.504	<0.001	/	/
Psychotic-like experience (PQB-21)								
PLE symptoms	2.81	3.25	0.329	<0.001	0.421	<0.001	0.412	<0.001
PLE distress	8.76	11.26	0.338	<0.001	0.407	<0.001	0.414	<0.001
Social anxiety symptoms (LSAS)								
Fear	20.16	13.00	0.484	<0.001	0.289	<0.001	0.329	<0.001
Avoidance	14.56	12.80	0.443	<0.001	0.254	<0.001	0.313	<0.001
Big Five Personality (BFI)								
Agreeableness	31.47	4.80	-0.326	<0.001	-0.242	<0.001	-0.328	<0.001
Conscientiousness	26.09	5.35	-0.151	<0.001	-0.084	<0.001	-0.186	<0.001
Extraversion	24.00	5.62	-0.496	<0.001	-0.084	<0.001	-0.244	<0.001
Neuroticism	25.58	5.69	0.348	<0.001	0.258	<0.001	0.323	<0.001
Openness	32.79	6.40	-0.040	0.024	0.107	<0.001	0.145	<0.001

SPQ-B, Schizotypal Personality Questionnaire-Brief; PQB-21, Prodromal Questionnaire-Brief; BFI, Big Five Inventory; LSAS, Liebowitz Social Anxiety Scale; N, 3186.

Linear regression was performed to examine the unique contribution from each SPQ-B subscale (**Table 3**). There was no significant multicollinearity between predictors: age (VIF = 1.004), sex (VIF = 1.007), Interpersonal subscale (VIF = 1.670), Cognitive-Perceptual subscale (VIF = 1.410), and Disorganization subscale (VIF = 1.772).

**Table 3:** Linear regressions showing the associations of the three SPQ-B subscales with PLEs (PQB-21) and social anxiety (LSAS).

	Psychotic-like experience (PQB-21)						Social anxiety symptoms (LSAS)					
Variables	PLE symptoms			PLE distress			Fear			Avoidance		
	*β*	*95% CI*	*ΔR²*	*β*	*95% CI*	*ΔR²*	*β*	*95% CI*	*ΔR²*	*β*	*95% CI*	*ΔR²*
Age	-0.086***	-0.136, -0.065		-0.081***	-0.450, -0.203		-0.060***	-0.419, -0.140		-0.019	-0.231, 0.053	
Sex	-0.043**	-0.484, -0.083		-0.017	-1.073, 0.320		0.149***	3.135, 4.715		0.120***	2.311, 3.916	
Schizotypal personality (SPQ-B)												
Interpersonal	0.059**	0.028, 0.139	0.002	0.076***	0.180, 0.566	0.003	0.427***	2.202, 2.641	0.109	0.386***	1.933, 2.378	0.089
Cognitive-Perceptual	0.274***	0.409, 0.533	0.053	0.250***	1.274, 1.705	0.044	0.077***	0.288, 0.776	0.004	0.051**	0.096, 0.591	0.002
Disorganization	0.236***	0.416, 0.587	0.032	0.241***	1.471, 2.066	0.033	0.027	-0.112, 0.563	0.000	0.050*	0.078, 0.763	0.001

*β*, Standardized coefficients beta; *ΔR²*, R² change; N, 3186; SPQ-B, Schizotypal Personality Questionnaire-Brief; PQB-21, Prodromal Questionnaire-Brief; LSAS, Liebowitz Social Anxiety Scale.

FDR-adjusted p-values. *p<.05, **p<.01, ***p<.001.

Interpersonal, Cognitive-Perceptual, and Disorganization subscales were significantly associated with PLE symptoms (*β* = 0.059; *β* = 0.274; *β* = 0.236) and distress (*β* = 0.076; *β* = 0.250; *β* = 0.241). Although all subscales have significant explanatory power on PLE outcomes, the Cognitive-Perceptual and Disorganization subscales had noticeably higher *R²* change than the Interpersonal subscale.

For social anxiety fear intensity, there were significant positive associations between Interpersonal (*β* = 0.427) and Cognitive-Perceptual (*β* = 0.077) subscales, but not Disorganization (*β* = 0.027). Similarly, social avoidance was positively associated with Interpersonal (*β* = 0.386) and Cognitive-Perceptual (*β* = 0.051) subscales, and Disorganization (*β* = 0.050). In terms of explanatory power, the Interpersonal subscale showed a greater contribution than the other two scales, especially on social avoidance (**Table 3**).

For the Big Five personality traits (**Table 4**), the Interpersonal subscale was significantly associated with all traits: agreeableness (*β*=−0.182), conscientiousness (*β*=−0.064), extraversion (*β*=−0.591), neuroticism (*β* = 0.219), and openness (*β*=−0.223). The Cognitive-Perceptual subscale was significantly associated with agreeableness (*β*=−0.068), extraversion (*β* = 0.171), neuroticism (*β* = 0.083), and openness (*β*=−0.089), but not conscientiousness (*β* = 0.021). The Disorganization subscale was significantly associated with agreeableness (*β*=−0.183), conscientiousness (*β*=−0.159), neuroticism (*β* = 0.144), and openness (*β* = 0.234), but not extraversion (*β* = 0.030). Agreeableness, extraversion, and neuroticism were best explained by the Interpersonal subscale, whereas Disorganization outperformed the others in explaining conscientiousness and openness.

**Table 4:** Linear regressions showing the associations of the three SPQ-B subscales with the Big Five personality traits (BFI).

	Big Five Personality (BFI)														
Variables	Agreeableness			Conscientiousness			Extraversion			Neuroticism			Openness		
	*β*	*95% CI*	*ΔR²*	*β*	*95% CI*	*ΔR²*	*β*	*95% CI*	*ΔR²*	*β*	*95% CI*	*ΔR²*	*β*	*95% CI*	*ΔR²*
Age	0.040*	0.013, 0.125		0.134***	0.192, 0.322		-0.058***	-0.176, -0.056		0.021	-0.022, 0.108		0.054**	0.047, 0.203	
Sex	0.027	-0.052, 0.577		0.011	-0.250, 0.486		0.005	-0.280, 0.396		0.146***	1.313, 2.048		0.045*	0.140, 1.020	
Schizotypal personality (SPQ-B)															
Interpersonal	-0.182***	-0.469, -0.294	0.020	-0.064**	-0.250, -0.046	0.002	-0.591***	-1.542, -1.355	0.209	0.219***	0.441, 0.645	0.029	-0.223***	-0.745, -0.501	0.030
Cognitive- Perceptual	-0.068***	-0.270, -0.076	0.003	0.021	-0.054, 0.173	0.000	0.171***	0.403, 0.611	0.021	0.083***	0.137, 0.365	0.005	0.089***	0.166, 0.438	0.006
Disorganization	-0.183***	-0.708, -0.439	0.019	-0.159***	-0.710, -0.396	0.014	0.030	-0.034, 0.254	0.001	0.144***	0.379, 0.693	0.012	0.234***	0.788, 1.163	0.031

*β*, Standardized coefficients beta; *ΔR²*, R² change; N, 3186. SPQ-B, Schizotypal Personality Questionnaire-Brief; BFI, Big Five Inventory.

FDR-adjusted p-values. *p<.05, **p<.01, ***p<.001.

## 4 Discussion

The current study was among the first to explore the factor structure of subclinical schizotypy traits using the SPQ-B in a large population-representative youth sample and its relationships with PLEs, social anxiety symptoms, and Big Five personality traits. Our first hypothesis was supported, CFA suggested that a three-factor model was a moderate-to-good fit by conventional criteria. Unique relationships between each subscale and outcome measures were observed. Our findings partially supported the second and third hypotheses that the three subscales were significantly associated with some but not all variables. Specifically, the subscales showed distinctive explanatory power: the Interpersonal subscale outperformed the other two in explaining social-related variables, but underperformed in explaining PLEs, conscientiousness, and openness.

As hypothesized, the three-factor model found in Western samples was replicable in the Hong Kong youth sample (44–46). This finding is also consistent with a Taiwanese college sample which found the three-factor model a more optimal fit than a one-factor model (38). However, we noted low factor loadings for two Cognitive-Perceptual items (items 4, 12) and overall low communalities for this factor. It is possible that the variance of these items was explained by more than the Cognitive-Perceptual factor alone, as the improved Cognitive-Perceptual psychometrics in sensitivity analysis suggested. For example, item 4 (“I am sometimes convinced that other people are able to guess what I think”) could be overlapping with social anxiety construct indexed by the Interpersonal factor or theory of mind distirubance indexed by the Disorganization factor. Indeed, some studies have noted that the SPQ-B item-factor relationship may not be clearly separated, such that several items may index more than one of the hypothesized factors (23, 47). This notion is also supported by the moderate correlation between the three subscales and their moderate internal consistencies, implying variability in the measurement of the intended construct in this sample.

Moreover, there could be cultural differences in the interpretation of items. Chinese traditional religions such as Buddhism & Taoism may influence subjective experience (48). This could manifest as superstition captured by item 12 (“It happened to me to have special experiences about astrology, premonition, unidentified flying objects, extrasensory perception, or the sixth sense”). The cultural variance in schizotypy subtypes indexed by the SPQ-B has been documented. A study found a different schizotypy factorial structure in Australia than in Chile and China, suggesting a lack of measurement invariance (49). Researchers have attributed the difference to the individualist-collectivist cultural difference and potential translation issue. Yet, even between Western countries, partial measurement invariance was observed (50). This could imply a low cross-cultural replicability of the SPQ-B structure, given the many factors that could affect the interpretation and answering of the measurement. The partial replicability of the factorial structure in the current Hong Kong sample also calls for further cross-cultural comparison to develop culturally-adaptive tools for screening (31).

The Cognitive-Perceptual and Disorganization factors were more strongly correlated with PLEs and related distress than the Interpersonal factor in this study, supported by a higher explanatory contribution. This is consistent with existing literature that highlights perceptual abnormalities and odd beliefs as correlates of PLE in the general population (51, 52). The presentation of disorganized cognition in high-risk youths or during the prodromal stage could also predict early neurobiological alterations, functioning decline, poorer outcomes, and poorer prognosis (53, 54). The relationship between Cognitive-Perceptual deficits, Disorganization, and PLE implies a neurocognitive underpinning of the overlap between schizotypy and schizophrenia (55, 56). This notion is supported in the identification of high-risk Chinese youths through screening schizotypy traits (7). For this reason, schizotypy should not be overlooked in the early detection of psychosis in youths.

Our findings were aligned with the expectation that schizotypy traits are associated with social outcomes, especially Interpersonal deficits. Interpersonal deficits can increase the proneness of social withdrawal and social stress, which are known predictors of psychosis transition (57–60). This can contribute to social barriers that limit the effect of environmental enrichment in building resilience. Some initial studies have found that enhancing social skills, social appraisals, and connectedness can reduce the risk of transitioning from schizotypy to schizophrenia (61–63). Profiling schizotypal traits may help identify distinctive needs among youths with high schizotypy for personalized interventions. Such that those who scored higher on Interpersonal deficits may benefit more from social intervention than those who scored higher on other deficits (64–66).

While schizotypy is referred to as a set of personality traits, our findings suggested distinctions between the three schizotypal dimensions and generic personality traits. In particular, the Cognitive-Perceptual and Disorganization subscales were not related to conscientiousness and extraversion, respectively, when Interpersonal deficits were accounted for. These findings concur with existing studies that found schizotypal traits associated with some but not all domains of the Big Five personality (30, 67). We added to the existing literature that not all dimensions of schizotypy were associated with conscientiousness and extraversion in a representative youth sample. Studies that explored the relationship between schizotypy and extraversion were varied, with some finding negative results (68), an association with one facet of extraversion (69), and both being on the same personality taxonomy (67). The inconsistent findings may be explained by the lack of significant association between Disorganization and extraversion in the current study sample, where high schizotypy, that is characterized predominantly by disorganized traits, may not be relevant to the inference of an extraverted personality. Future studies should therefore take into consideration the different roles of individual schizotypy domains in normal personality traits.

The findings also replicated the relationship between schizotypy and openness to experience (27, 70, 71). Positive schizotypy can represent a maladaptive high-end of openness (26). Independently, individuals may be able to experience what was considered a “healthy schizotypy” when positive schizotypal traits are successfully integrated into a meaningful framework (12). However, disorganized cognition, a negative evaluation of the experience, may induce distress and affect well-being (12). This could hold clinical implications for early detection and intervention, where schizotypy can interact with normal personality dimensions to predispose youths to schizophrenia (27, 67, 72). Identifying maladaptive changes to interpretations of experience could become an important early sign to identify the risk of transition.

The current study is not without limitations. First, the Cognitive-Perceptual subscale exhibited suboptimal psychometric properties, with multiple items showing weak factor loadings and low communalities. This measurement issue may have attenuated observed relationships with external variables, potentially underestimating this construct’s true differential predictive validity. Future research should consider item refinement or development of improved indicators for the Cognitive-Perceptual dimension. A minimal exclusion criterion in the current sample could also introduce heterogeneity, where the effects of other possible confounding variables, such as affective symptoms, neurocognitive ability, or substance use, could affect how generalizable results are to specific populations. However, such inclusivity is not uncommon across epidemiological studies. While the sample might be over-represented by youths with low schizotypy and PLEs, the recruitment method generates a population-representative sample. This provided additional value to a conventional evaluation of schizotypy structure. Together, the psychometric properties of the Cognitive-Perceptual subscale and the heterogeneity of the recruited sample should be considered when interpreting the results.

## 5 Conclusion

The current study examined a three-factor model and respective loading of self-reported subclinical schizotypy traits dimensions among Hong Kong youths. Our findings support the conceptual notion that subclinical schizotypy is a multifaceted structure. Similar to previous studies, some items were not distinctly separated between factors due to the strong association between the subconstructs. Nevertheless, the hypothesized factors were significantly associated with PLEs, social anxiety, and Big Five personality traits. Specifically, social outcomes were better explained by Interpersonal deficits, whereas Cognitive-Perceptual deficits and Disorganization had higher explanatory power on PLE outcomes. The findings concur with the existing body of work that calls for attention to social factors in schizotypy and transition to schizophrenia. Schizotypal profiles may be identified for targeted interventions to build resilience in youths with specific deficits.

## Data Availability

The de-identified raw data supporting the conclusions of this article will be made available by the authors upon reasonable request, without undue reservation.

## Supplementary material

Supplementary Table 1 Participant demographics.

Supplementary Table 2 Standardized loading and factor correlation of the four-factor SPQ-B, model using CFA; SPQ-B, Schizotypal Personality Questionnaire-Brief; N, 3186; ***p < .001 # Paranoid Ideation items are expected to load on both Cognitive-Perceptual and Interpersonal factors.
