# Supplementary material for: The unique contribution of Schizotypal personality subscales to psychotic-like experiences and social-personality factors in Hong Kong community youths
Source: Front Psychiatry. 2026 Mar 3;17:1590707. doi: 10.3389/fpsyt.2026.1590707 (PMC12992260; doi:10.3389/fpsyt.2026.1590707)
Supplement: Supplementary Table 1 — Participant demographics. [file Table1.docx]

## **Supplementary Materials I**

| **Supplementary Table 1. Participant demographics.** | | |  |  |  |
| --- | --- | --- | --- | --- | --- |
|  | **Included sample (n = 3186)** | | **Excluded sample (n = 154)** | | ***p*** |
|  | **Mean** | **SD** | **Mean** | **SD** |  |
| Age | 19.84 | 2.79 | 19.43 | 2.97 | 0.074 |
|  | **N** | **%** | **N** | **%** |  |
| Sex (female) | 1854 | 58.2 | 1332 | 41.8% | 0.008 |
| Ethnicity |  |  |  |  |  |
| Chinese | 3110 | 97.6 | 144 | 93.5 | <0.001 |
| Indonesian | 1 | 0 | 0 | 0 |  |
| Philipian | 4 | 0.1 | 1 | 0.6 |  |
| Caucasian | 6 | 0.2 | 0 | 0 |  |
| Indian | 16 | 0.5 | 2 | 1.3 |  |
| Pelastinian | 1 | 0 | 2 | 1.3 |  |
| Nepali | 5 | 0.2 | 1 | 0.6 |  |
| Other Asians | 8 | 0.3 | 0 | 0 |  |
| Other ethnicity | 12 | 0.4 | 3 | 1.9 |  |
| Mix | 23 | 0.8 | 1 | 0.6 |  |
| First language |  |  |  |  |  |
| Cantonese | 2965 | 93.1 | 139 | 90.3 | 0.169 |
| Others | 218 | 6.8 | 15 | 9.7 |  |
| Education level |  |  |  |  |  |
| Primary | 19 | 0.6 | 1 | 0.6 | 0.133 |
| Form 1-3 | 320 | 10 | 21 | 13.6 |  |
| Form 4-5 | 447 | 14 | 31 | 20.1 |  |
| Form 6-7 | 1326 | 41.6 | 48 | 31.2 |  |
| Diploma | 87 | 2.7 | 6 | 3.9 |  |
| Higher diploma | 325 | 10.2 | 13 | 8.4 |  |
| Top-up degree | 23 | 0.7 | 0 | 0 |  |
| Undergraduate | 586 | 18.4 | 32 | 20.8 |  |
| Postgraduate | 53 | 1.7 | 2 | 1.3 |  |

## **Supplementary Materials II**

| **Supplementary Table 2. Standardised loading and factor correlation of the four-factor SPQ-B model using CFA.** | | | | |
| --- | --- | --- | --- | --- |
|  |  | Factor loading | | *h^2^* |
| Items | | *λ* | 95% CI |  |
| Interpersonal | |  |  |  |
| 1. | Sometimes other people think that I am detached or distant | 0.730 | 0.694, 0.766 | 0.533 |
| 11. | I don’t feel at all at ease when I am around people I don’t know | 0.613 | 0.574, 0.653 | 0.376 |
| 18. | I have the feeling that I can’t be close to people | 0.852 | 0.826, 0.878 | 0.725 |
| 21. | I don’t feel comfortable when I talk to people I don’t know well | 0.810 | 0.780, 0.839 | 0.655 |
| 22. | I tend to keep my feelings to myself | 0.665 | 0.624, 0.706 | 0.422 |
| Cognitive-Perceptual | |  |  |  |
| 2. | I happen to feel an unseen force or presence around me | 0.648 | 0.593, 0.702 | 0.419 |
| 4. | I am sometimes convinced that other people are able to guess what I think | 0.385 | 0.331, 0.439 | 0.148 |
| 5. | It happens to me that certain objects or ordinary situations have a special significance for me | 0.655 | 0.612, 0.698 | 0.429 |
| 10. | When I go shopping I have the feeling that people notice me | 0.673 | 0.628. 0.718 | 0.453 |
| 12. | It happened to me to have special experiences about astrology, premonition, unidentified flying objects, extrasensory perception, or the sixth sense | 0.398 | 0.345, 0.451 | 0.158 |
| 16. | Sometimes, I'm suddenly distracted by distant sounds to which usually I don't pay much attention. | 0.725 | 0.683, 0.768 | 0.526 |
| Disorganization | |  |  |  |
| 3. | Sometimes other people comment on my behavior particularities or my unusual habits | 0.669 | 0.628, 0.711 | 0.448 |
| 6. | Some people believe I am very odd/bizarre | 0.830 | 0.803, 0.857 | 0.689 |
| 8. | Some people consider my way of speaking vague or not too clear | 0.774 | 0.743, 0.805 | 0.598 |
| 13. | I sometimes use words in an unusual way | 0.593 | 0.539, 0.647 | 0.352 |
| 19. | I am a strange or unusual person | 0.799 | 0.772, 0.826 | 0.639 |
| 20. | It’s difficult for me to make other people understand what I want to say | 0.741 | 0.705, 0.776 | 0.549 |
| Paranoid Ideation # | |  |  |  |
| 7. | I feel that I must be vigilant even with my friends | 0.636 | 0.598, 0.674 | 0.405 |
| 14. | I think it’s better that people don’t know too much about me | 0.723 | 0.691, 0.755 | 0.523 |
| 15. | I tend to stay withdrawn when I am in social situations | 0.812 | 0.784, 0.840 | 0.659 |
| 9. | It often happens to me to see hidden threats or derogatory remarks in what other people say or do | 0.656 | 0.619, 0.692 | 0.430 |
| 17. | I often must be vigilant for other people not to take advantage of me | 0.481 | 0.439, 0.523 | 0.231 |
| Factor Correlations | | *r* | *95% CI* |  |
|  | Cognitive-Perceptual x Interpersonal | 0.525 | 0.477, 0.574*** |  |
|  | Cognitive-Perceptual x Disorganization | 0.720 | 0.680, 0.760*** |  |
|  | Cognitive-Perceptual x Paranoid Ideation | 0.748 | 0.773, 0.788*** |  |
|  | Interpersonal x Disorganization | 0.803 | 0.773, 0.833*** |  |
|  | Interpersonal x Paranoid Ideation | 0.874 | 0.845, 0.903*** |  |
|  | Disorganization x Paranoid Ideation | 0.823 | 0.793, 0.854*** |  |
| *Note.* SPQ-B = Schizotypal Personality Questionnaire-Brief. N = 3186. ****p*<.001  # Paranoid Ideation items are expected to load on both Cognitive-Perceptual and Interpersonal factors. | | | | |
